# Extraction Effects on Roselle Functionalities: Antioxidant, Antiglycation, and Antibacterial Capacities

**DOI:** 10.3390/foods13142172

**Published:** 2024-07-09

**Authors:** Ying-Jang Lai, Yi-Chan Chiang, Yi-Syuan Jhan, Tuzz-Ying Song, Ming-Ching Cheng

**Affiliations:** 1 Department of Food Science, National Quemoy University, Kinmen 892, Taiwan; 2 Department of Food Science and Biotechnology, National Chung Hsing University, Taichung 402, Taiwan; 3 Department of Food and Nutrition, Providence University, Taichung 433, Taiwan; 4 Department of Food Science and Technology, Central Taiwan University of Science and Technology, Taichung 406, Taiwan; 5 Department of Health Food, Chung Chou University of Science and Technology, Changhua 515, Taiwan; 6 Department of Medicinal Botanicals and Foods on Health Applications, Da-Yeh University, Changhua 515, Taiwan; 7 Department of Food Science and Technology, Hungkuang University, Taichung 433, Taiwan

**Keywords:** roselle anthocyanins, extraction methods, functional properties, antioxidant activity, antiglycation capacity

## Abstract

This study investigated the effects of certain roselle (*Hibiscus sabdariffa* Linnaeus) extraction methods on various functional properties, including the antioxidant and antiglycation capacities and bacterial growth inhibition. Roselle anthocyanins were extracted using water and ethanol solvents at different temperatures and concentrations. The results revealed that the extraction rate increased with higher temperatures and ethanol concentrations (*p* < 0.05). Ethanol extracts exhibited higher total organic acid and total anthocyanin contents compared to water extracts, while water extracts showed higher total saccharide, total polyphenol, and total flavonoid contents (*p* < 0.05). Furthermore, the water extracts demonstrated superior Trolox equivalent antioxidant capacity (TEAC) values, while the ethanol extracts exhibited better 2,2-diphenyl-1-picrylhydrazyl (DPPH) radical scavenging ability, antiglycation capacity, and bacterial growth inhibition. A Pearson correlation analysis revealed strong associations between specific components and functional properties, such as a positive correlation between the total anthocyanin content and antiglycation capacity (*R*^2^ = 0.9862). A principal component analysis and agglomerative hierarchical clustering highlighted distinct clusters of water and ethanol extracts, indicating solvent-dependent variations in functional properties. This study assessed roselle extraction models for antioxidant, antiglycation, and antibacterial activities, which could be used for the development of functional alcoholic or non-alcoholic beverages.

## 1. Introduction

In contemporary society, the demand for health and nutritional supplements has prompted sustained interest in diet and health products. An increasing number of people are focusing on the use of bioactive compounds present in natural plants for therapy or the alleviation of oxidative stress [1]. Physiological imbalances induced by oxidative stress may lead to various diseases, such as nephritis, diabetes, and cerebrovascular and cardiovascular diseases. As the oxidative stress increases, it further leads to aging, cancer, and problems with the immune and digestive systems [2,3]. The research on ingredients with antiglycation capacities is increasingly garnering attention. When one’s insulin receptor sensitivity decreases, their blood glucose level increases significantly, initiating a series of glycation reactions that disrupts the collagen fibers in the skin, leading to aging and loss of elasticity. Antiglycation activity has been reported to slow the rate of blood glucose elevation by reducing the formation of advanced glycation end products (AGEs), thereby aiding in the prevention of metabolic diseases such as diabetes [4,5,6]. Moreover, it has been found to be beneficial for cardiovascular health and neurological function [7,8]. Similar to antiglycation activity, antioxidant activity is widely researched and considered crucial for human health. Antioxidants neutralize free radicals and reduce oxidative damage, thereby aiding in the prevention of inflammation, cellular aging, and the onset of chronic diseases [9,10].

Among numerous plants, roselle (*Hibiscus sabdariffa* Linnaeus) is widely popular due to its unique composition of bioactive compounds, such as saccharides, organic acids, and polyphenols. Anthocyanins, especially those abundant in roselle, are water-soluble, non-toxic natural pigments derived from the secondary metabolic pathway of flavonoids [11,12]. Previous studies have indicated that roselle anthocyanins are potent antioxidants. The chemical structure of anthocyanins consists of two aromatic rings (A and B rings) and an oxygen-containing heterocycle (C ring). As there are no electrons in the C ring, it effectively absorbs unpaired electrons, exhibiting characteristics such as scavenging reactive oxygen species, terminating oxidative chain reactions, and reducing harm to the human body [13,14]. Roselle anthocyanins have been proven to possess strong antioxidant, anti-inflammatory, LDL-lowering, blood-pressure-lowering, lipid-lowering, antiatherosclerotic, antidiabetic, and anticancer effects, significantly reducing the incidence of metabolic syndrome [15,16,17]. Previous animal experiments have also indicated their ability to reduce blood glucose, body weight, food intake, urine volume, and fecal volume levels in diabetic rats [18]. Anthocyanins also exhibit significant antibacterial properties, making them promising natural health food ingredients [19]. According to a study by Liu et al. [20], the water and ethanol extracts of roselle anthocyanins can inhibit the growth of pathogenic bacteria, such as *Staphylococcus aureus*, *Streptococcus pneumoniae*, *Escherichia coli* O157:H7, *Listeria monocytogenes*, and *Salmonella* DT104 [21,22,23]. However, the stability and concentration of anthocyanins can be influenced by various external factors [24,25]. Although roselle anthocyanins have been extensively studied, this study aimed to rapidly evaluate the extraction models for different ethanol concentrations and temperatures by evaluating their antioxidant, antiglycation, and antibacterial activities with a principal component analysis (PCA) for improved functional roselle extraction selectivity and the development of related products, such as alcoholic and non-alcoholic beverages.

## 2. Materials and Methods

### 2.1. Materials and Chemicals

**Figure 1:**
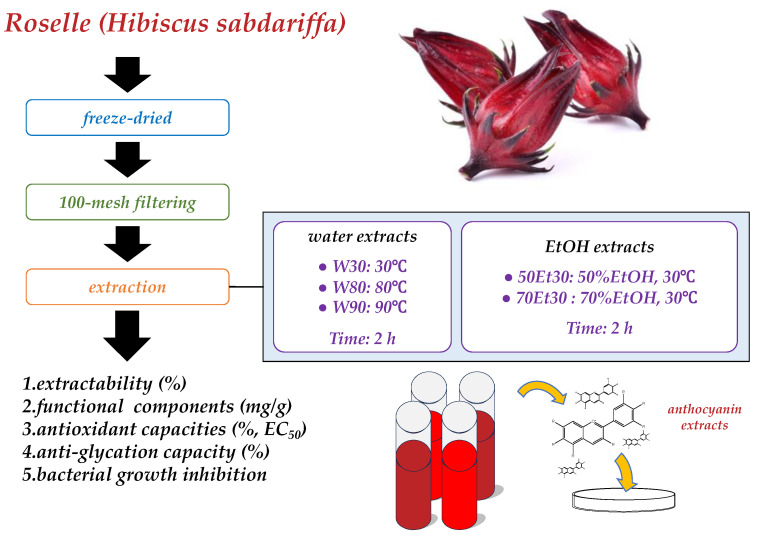
Experimental structure.

Fresh roselles were purchased from Biomed Herbal Research Co., Ltd. (Taichung, Taiwan). For each sample, a similar weight was selected (3.0 ± 0.5 g), washed, freeze-dried, milled to a powder, filtered over 100 mesh filters, and stored at −18 °C for analysis. This study divided the roselles into five groups (Figure 1). All chemical reagents used in this study were of the analytical grade and were purchased from Sigma-Aldrich Co. (St. Louis, MO, USA).

### 2.2. Roselle Anthocyanin Extraction

For this extraction system, the same extraction ratio was used with distilled water or ethanol (1:20, *w*/*v*); different temperatures were used for the water extracts (30, 80, and 90 °C), and different concentrations were used for developing ethanol extracts (50% and 70% ethanol). All extractions were stirred and extracted for 2 h. Subsequently, we used 90 mm Ø qualitative filter paper (Whatman 1001-090, Whatman International Ltd., Maidstone, UK) and a rotary evaporator at 50 °C (Hei-Vap Expert Control, Heidolph Persia Co., Ltd., Tehran, Iran). The roselle extract was obtained after being freeze-dried for 3 days. Following the method of Li et al. [10], the extractability was calculated using Equation (1):

Extractability (%) = [freeze-dried extract (g)/extract (g)] × 100(1)

### 2.3. Functional Components

#### 2.3.1. Total Saccharides and Total Organic Acids

The total saccharide (TS) content and total organic acid (TO) content were determined using the method used by Younis [26].

#### 2.3.2. Total Anthocyanins

The total anthocyanin (TA) content was measured using the method used by Lu et al. [27] with Equation (2), where A_530_ and A_657_ represent the absorbance measured under wavelengths of 530 and 657 nm, respectively; V, ε, and W represent the extractant volume (mL), absorption coefficient (31.6 mM^−1^ cm^−1^), and sample weight (g), respectively:

Total anthocyanins (μmol/g DW) = [(A_530_ − 0.33 × A_657_) × V]/(ε × W) (2)

#### 2.3.3. Total Polyphenols and Total Flavonoids

The total polyphenol (TP) content and total flavonoid (TF) content were determined using the method used by Hsu et al. [28] with gallic acid and quercetin, respectively, as the standards.

### 2.4. Antioxidant Capacities

The measurements of the 2,2-diphenyl-1-picrylhydrazyl (DPPH) radical scavenging and Trolox equivalent antioxidant capacity (TEAC) rates were performed using the method used by Li et al. [29] with Equations (3) and (4), respectively. The absorbance values measured under wavelengths of 518 and 734 nm are represented as A_518_ and A_734_, respectively:

DPPH radical scavenging (%) = (A_518_ of sample − A_518_ of control)/(A_518_ of sample) (3)

TEAC (%) = 1 − [(A_734_ of sample − A_734_ of control)/(A_734_ of blank)] (4)

### 2.5. Antiglycation Capacity

Following a modified version of the method used by Wu and Yen [30], we used 1% phosphate-buffered saline (pH 7.4) containing fetal bovine serum albumin (50 mg/mL) and 0.8 M glucose. The total volume was quantified and filtered through a 0.22 μm filter. We divided the samples into experimental, blank, and control groups and then incubated them at 37 °C in an incubator. Using a fluorescence spectrophotometer (model FLx800, BioTek Instruments, Inc., Winooski, VT, USA) with excitation at 330 nm and emission at 410 nm, we measured the absorbance in triplicate after one week of incubation to calculate the antiglycation activity levels of different roselle extracts.

### 2.6. Bacterial Growth Inhibition

#### 2.6.1. Preparation of Culture Medium, LB Agar Plates, and LB Agar Overlay

For the medium configuration, the LB culture medium powder, sodium chloride (NaCl), peptone, and yeast extract were dissolved in deionized water. The solution was then aliquoted into test tubes (5 mL/medium). The tubes were sealed with aluminum foil. After sterilization at 121 °C for 15 min using an autoclave, the medium was cooled to room temperature and stored at 4 °C for later use in liquid culture. For the LB agar plate medium configuration, the LB culture medium powder, NaCl, peptone, yeast extract, and agar were dissolved in deionized water (1.5%) were placed in serum bottles. The serum bottles were sealed with aluminum foil and sterilized at 121 °C for 15 min using an autoclave. After cooling to room temperature, 10 mL of the agar medium was dispensed into sterile plastic Petri dishes and stored at 4 °C to prepare the LB agar plates for bacterial culture. For the LB agar overlay plate medium configuration, the LB culture medium powder, NaCl, peptone, yeast extract, and agar were dissolved in deionized water (0.25%). The serum bottles were sealed with aluminum foil and sterilized at 121 °C for 15 min using an autoclave. This LB agar overlay was prepared for the bacterial cultures.

#### 2.6.2. Bacterial Subculture

Following a modified version of the method used by Chou et al. [31], the bacterial cultures were transferred under aseptic conditions to an aseptic workspace. Two microorganisms, *E. coli* and *S. aureus*, were inoculated to a fresh culture medium and mixed thoroughly using a sterile inoculating loop. The cultures were then incubated at 37 °C for 24 h.

#### 2.6.3. Minimum Inhibitory Concentration (MIC) and Minimum Bactericidal Concentration (MBC)

Following a modified version of the method used by Chavan and Nadanathangam [32], we mixed the bacterial solution evenly and measured 1 mL of the solution at 640 nm. We diluted the bacterial solution to the appropriate turbidity and added different concentrations of roselle extracts in an incubator (100 rpm, 37 °C, 24 h). Then, we observed the absorbance value at 640 nm to determine the bacterial inhibition of the roselle extracts. Under a specific concentration of roselle extracts, the growth of bacteria means that the concentration reaches only the antibacterial level in the MIC test. The MBC is the minimum concentration in a Petri dish at which no bacteria grow.

### 2.7. Statistical Analysis

The data obtained in the experiment were statistically analyzed using SPSS 18.0 software (IBM Co., Armonk, NY, USA). The experimental results are presented as the mean ± standard deviation (mean ± SD) (*n* = 3), and Duncan’s multiple range test was employed to determine differences among experimental groups. Statistical significance was considered to be *p* < 0.05. The principal component analysis (PCA), agglomerative hierarchical clustering (AHC), and Pearson correlation analysis results were exported with XLSTAT software (Version 2024.2.2, Addinsoft Co., New York, NY, USA).

## 3. Results and Discussion

### 3.1. Functional Composition of Roselle Extracts

The freeze-dried powder of Taiwan roselle plants was crushed and filtered over 100 mesh filters, then 100 g samples were weighed for extraction with water at different temperatures (30, 80, and 90 °C) and ethanol at different concentrations (50% and 70%). Table 1 shows that the extractability values of the water extracts at different temperatures (30, 80, and 90 °C) were 46.0 ± 4.4%, 49.3 ± 2.7%, and 49.2 ± 2.8%, respectively. As the temperature increased, the extraction rate showed an increasing trend. The ethanol extraction rates were 45.8 ± 3.7% and 42.2 ± 5.9%, respectively, at the different concentrations (50% and 70%, respectively). During the process of extracting roselle anthocyanins using water and ethanol, the extraction rate was dependent on the difference in ethanol concentration and water extraction temperature, as a previous study found [33]. When the polarity of the extraction solvent decreases, the extractability tends to decrease. Related studies noted that when *Scutellaria baicalensis* was extracted with different solvents, the polarity of the solvent affected the extractability and needed to be considered to obtain the appropriate extraction results [34].

Table 1 shows that the TS contents of the water extractions under different conditions ranged from 98.0 to 124.1 mg/g, among which the TS contents of the 90 °C water extracts were the highest (124.1 ± 3.6 mg/g), followed by the 80 °C water extracts (100.8 ± 0.4 mg/g) and the 30 °C water extracts (98.0 ± 1.6 mg/g). The TS content of the 50% ethanol extracts was 46.4 ± 0.5 mg/g, and no TS was detected in the 70% ethanol extracts. This phenomenon might have occurred because the polysaccharides have a polyol structure, which is weakly acidic. When ethanol is used as the extraction solvent, different concentrations influence the TS precipitate, and polysaccharides are mutually soluble, which greatly reduces the extraction efficiency. When the ethanol concentration increases, the polysaccharide content decreases because it has multiple hydroxyl groups; when the ratio of ethanol reaches a certain level, the hydroxyl groups dissolve together [35]. In summary, the water extracts had higher TS contents than the ethanol extracts.

The TO contents of the water extracts under different conditions ranged from 32.3 ± 0.1 to 34.1 ± 0.6 mg/g. The 80 °C water extracts had the highest contents, followed by the 90 and 30 °C extracts. In contrast, the TO contents of the ethanol extracts ranged from 41.4 ± 0.1 to 44.1 ± 0.4 mg/g, while those of the 70% ethanol extracts were higher than the 50% ethanol extracts (Table 1). The TA contents of the water extracts under different conditions ranged from 0.8 ± 0.0 to 1.1 ± 0.1 mg/g. The 30 °C water extracts had the highest contents, followed by the 80 and 90 °C extracts. In contrast, the TA contents of the ethanol extracts ranged from 1.8 ± 0.0 to 2.1 ± 0.1 mg/g, while those of the 50% ethanol extracts were higher than the 70% ethanol extracts (Table 1). As the concentration of ethanol increases, the TA content decreases slightly. Previous studies have indicated that higher extraction temperatures reduce the content of anthocyanins because they accelerate the decomposition of the chemical structure [36]. In addition, having an appropriate ethanol concentration affects the dissolution of anthocyanins and other phenolics [37]. In this case, the 50% ethanol extract was better than others. Past studies have indicated that adding ethanol can increase the color intensity and total acid content of a sample during extraction or storage [38]. In other words, appropriate ethanol extraction can increase the pigment stability [39]. The co-coloration effect not only affects the degree of anthocyanin polymerization but also prevents its oxidation [40]. In addition, co-coloration with phenolic acids and anthocyanins changes the color intensity during extraction [24].

Table 1 shows that the TP contents of the water extracts under different conditions ranged from 10.5 ± 0.2 to 13.0 ± 0.2 mg/g. The 90 °C water extracts had the highest contents, followed by the 80 and 30 °C extracts. In contrast, the TP contents of the ethanol extracts ranged from 5.8 ± 0.3 to 6.0 ± 0.5 mg/g, while those of the 70% ethanol extracts were higher than the 50% ethanol extracts. The TF contents of water extracts under different conditions ranged from 8.8 ± 0.3 to 11.4 ± 0.2 mg/g. The 90 °C water extracts had the highest contents, followed by the 80 and 30 °C extracts. In contrast, the TF contents of the ethanol extracts ranged from 4.9 ± 0.6 to 5.5 ± 0.2 mg/g; the TF contents of the 70% ethanol extracts were higher than the 50% ethanol extracts. Overall, the water extracts contained significantly higher TP and TF contents than the ethanol extracts (*p* < 0.05). According to previous studies, the TP contents of fragrant sunflower, which belongs to the same genus as roselle, showed similar results; room temperature water extracts (3.74 mg/g) had a higher TP content than hot water extracts (1.73 mg/g), which had a higher TP content than 80% ethanol extracts (1.56 mg/g). In addition, the TF contents of ambrette, which belongs to the same genus as roselle, showed similar results; room temperature water extracts (0.1 mg/g) had a higher TP content than hot water extracts (0.2 mg/g), which had a higher TP content than 80% ethanol extracts (0.3 mg/g) [14].

### 3.2. Antioxidant Capacities of Roselle Extracts

The TEAC was used to evaluate the antioxidant activity of the roselle extracts. The higher the value, the better the free radical scavenging ability. The TEAC values of water extracts under different conditions ranged from 115.9 ± 4.5 to 132.0 ± 3.4 µM. The 90 °C water extracts had the highest content, followed by the 30 and 80 °C extracts. In contrast, the TEAC contents of the ethanol extracts ranged from 93.9 ± 3.5 to 102.6 ± 3.4 µM, while those of the 70% ethanol extracts were higher than the 50% ethanol extracts (Table 2). Water extraction was better than ethanol extraction, which is consistent with previous studies. One study found that the TEAC value for an aqueous extract of elderberry fruits (1850.0 µM) was greater than that of a 70% ethanol extract (1520.0 µM) [41]. This phenomenon was due to the antioxidant properties of the total polyphenols and phenolic derivatives in the sample; samples will show different antioxidant properties mainly based on the number and position of the OH groups in their molecular structures [42]. Therefore, the polyphenol content is positively correlated with antioxidants [43].

**Figure 2:**
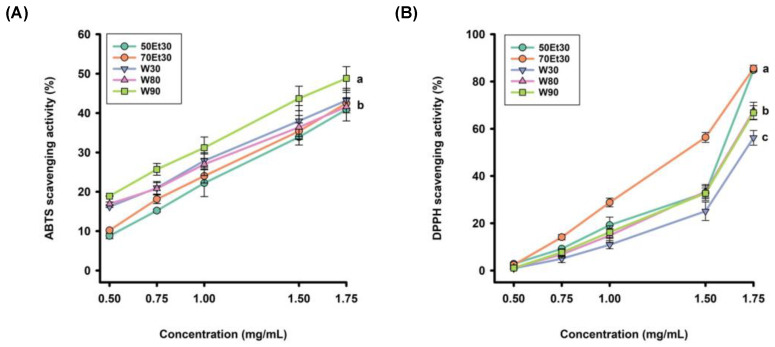
The antioxidant activity rates of the roselle extracts: (**A**) ABTS scavenging activity rates; (**B**) DPPH scavenging activity rates. Each value is expressed as the mean ± standard deviation (*n* = 3). Values (a–c) with different letters within the same column indicate significant differences (*p* < 0.05). Abbreviations: W30, W80, and W90 represent the sample extracted with water at 30, 80, and 90 °C, respectively; 50Et30 and 70Et30 represent the samples extracted with 0% and 70% ethanol, respectively.

The DPPH radical scavenging level can also be used to evaluate the antioxidant activity of roselle extracts. The free radical scavenging rates were observed in the extracts with different concentrations. Figure 2 shows that the scavenging rate of the ethanol extracts was higher than those of the 80 and 90 °C water extracts, while the 30 °C water extracts had the lowest clearance rate. Furthermore, the 70% ethanol extracts had the best DPPH radical scavenging ability, which increased with increasing concentrations. The EC_50_ values of the DPPH radical scavenging rates in water extracts under different conditions ranged from 28.7 ± 2.2 to 33.8 ± 2.3 µg/g. The 90 °C water extracts had the highest scavenging ability, followed by the 90 and 80 °C extracts. In contrast, the scavenging rates of the ethanol extracts ranged from 21.1 ± 1.8 to 26.2 ± 1.7 µg/g, while those of the 50% ethanol extracts were higher than the 70% ethanol extracts (Table 2). As DPPH is a hydrophobic phase-free radical, it can be completely soluble in ethanol or methanol but relatively insoluble in water. It will change with the pH and time and is relatively stable over a pH range of 5.0–5.6 [44]. Overall, the ethanol extracts showed better DPPH free radical scavenging ability than the water extracts, with the 70% ethanol extract being the best. In summary, the 90 °C water and 70% ethanol extracts had the best TEAC and DPPH EC_50_ values, respectively.

### 3.3. Antiglycation Capacity of Roselle Extracts

**Figure 3:**
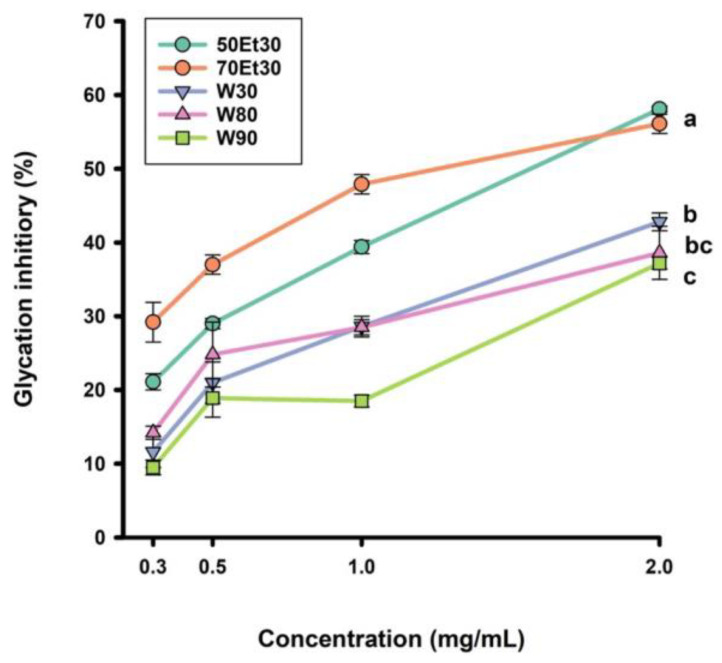
The glycation inhibitory values of roselle extracts. Each value is expressed as the mean ± standard deviation (*n* = 3). Values (a–c) with different letters within the same column indicate significant difference (*p* < 0.05). Abbreviations: W30, W80, and W90 represent the samples extracted with water at 30, 80, and 90 °C, respectively; 50Et30 and 70Et30 represent the samples extracted with 0% and 70% ethanol, respectively.

The antiglycation capacity of the roselle extracts at different concentrations is shown in Figure 3. The four concentrations were 0.3, 0.5, 1.0, and 2.0 mg/mL, and the glycation inhibitory range was 9.5–42.8%. For the 2.0 mg/mL roselle extracts, the glycation inhibitory capacity rates of the 30, 80, and 90 °C water extracts reached 42.8%, 38.6%, and 37.2%, respectively. However, the glycation inhibitory values of the ethanol extracts ranged from 56.1 to 58.1% (*p* < 0.05). As the concentration of roselle extract increased, the ability to inhibit glycation was significantly improved (*p* < 0.05). As in a previous study, the ethanol extracts had higher glycation inhibitory values than the water extracts. Low anthocyanin concentrations can block the adsorption of sugar molecules on proteins, thereby achieving inhibition [45]. According to past studies, ethanol extracts of many medicinal plants have the ability to inhibit microvascular diseases, complications of diabetes, aging, neurodegeneration, and other diseases [46,47]. As ethanol is more hydrophobic than water, it can extract more polyphenols from plants. These polyphenols can stop the formation of AGEs in the blood, lower the amount of glycoproteins, slow vascular damage [18], and decrease risk factors for metabolic syndrome [4]. We will further investigate the unknown phenolic compounds of roselle extracts that inhibit AGEs in future studies.

### 3.4. Bacterial Growth Inhibition of Roselle Extracts

The antibacterial ability rates of the different roselle extracts are shown in Table 3. The water extracts could not inhibit *E. coli* growth, and the 80 and 90 °C water extracts showed no antibacterial activity against *S. aureus*. Only the 30 °C water extracts showed antibacterial and bactericidal abilities; their MIC and MBC rates were 0.6 and 1.2 mg/mL, respectively. The 50% and 70% ethanol extracts showed good inhibitory effects; the MICs for *E. coli* were 0.9 and 1.0 mg/mL, respectively, and the MBC and MIC were the same. The MIC for inhibiting *S. aureus* was 0.6 mg/mL, while the MBCs were 1.0 and 1.2 mg/mL, respectively. We found that the ethanol extracts of roselle could kill Gram-negative bacteria such as *E. coli* (G−) and stop Gram-positive bacteria such as *S. aureus* (G+) from growing. Overall, the ethanol extracts were significantly better than the water extracts at inhibiting bacterial growth (*p* < 0.05). If the MIC and MBC values are close, then an extract has both antibacterial and bactericidal capabilities at the same concentration. At different concentrations, the MIC and MBC values are used to indicate that the extract has progressive bacteriostatic and bactericidal abilities [48,49]. This result is consistent with previous results reported in many plants; that is, the MIC values of methanol and ethanol extracts are better than those of water extracts, and the antibacterial effect of natural antibacterial ingredients on Gram-positive bacteria is better than that on Gram-negative bacteria. Natural antibacterial ingredients mainly include phenolic compounds, terpenes, aldehydes, and ketones, all of which have both antibacterial and bactericidal abilities [50]. In addition, phenolic compounds can inhibit the aggregation of *S. aureus* colonies, reduce the number of bacteria, and achieve bacteriostatic effects [51,52].

### 3.5. Correlation Analysis, Principal Component Analysis, and Agglomerative Hierarchical Clustering Analysis of Roselle Extracts

**Figure 4:**
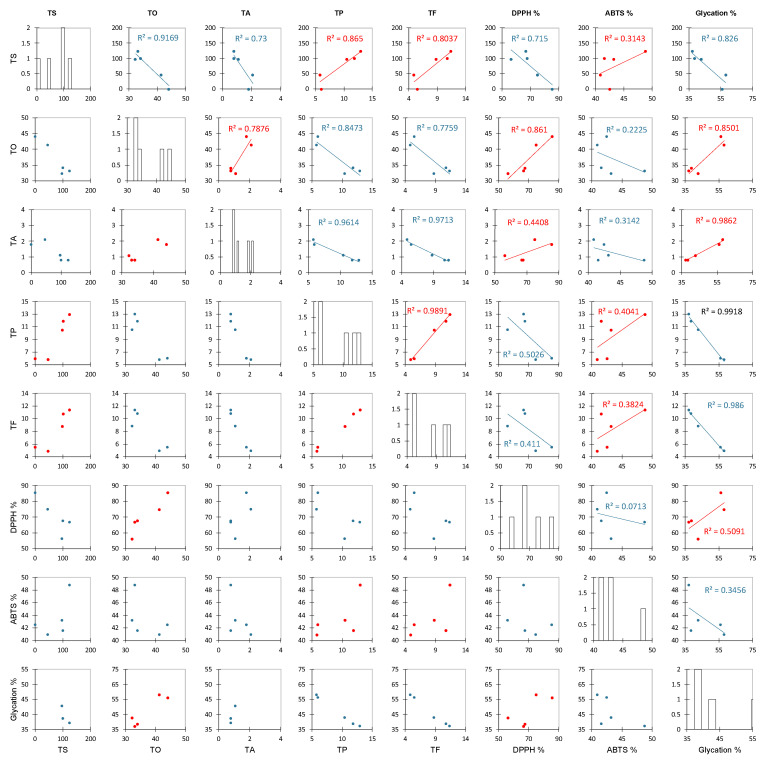
The Pearson correlation scatter plots of roselle extracts. Abbreviation: TS, TO, TA, TP, TF, DPPH%, ABTS%, and glycation% represent the total saccharides, total organic acids, total anthocyanins, total polyphenols, total flavonoids, DPPH radical scavenging for 1.75 mg/mL of extract, ABTS scavenging activity for 1.75 mg/mL of extract, and antiglycation capacity.

Before analyzing all of the collected data, we abbreviated the names of different factors. Here, TS, TO, TA, TP, TF, DPPH %, ABTS %, and glycation % represent the total saccharides, total organic acids, total anthocyanins, total polyphenols, total flavonoids, DPPH radical scavenging activity at 1.75 mg/mL of extract, ABTS scavenging activity of at 1.75 mg/mL of extract, and antiglycation capacity, respectively. Figure 4 shows the results of a Pearson correlation analysis between these factors. The closer the *R*^2^ of the positive and negative correlations is to 1, the stronger the correlation. Ascorbic acid and citric acid, which are the main components of TO in roselle, showed a strong positive correlation (0.8610) with scavenging DPPH free radicals but a weak negative correlation (0.2225) with ABTS free radical scavenging [35]. Due to the different solubility rates of free radicals, DPPH free radicals dissolve towards the organic phase while ABTS free radicals dissolve towards the aqueous phase, so the ability of organic acids to scavenge DPPH free radicals is better than that for ABTS free radicals [53,54]. A previous study mentioned that organic acids of roselle inhibit Gram-negative bacteria more effectively than Gram-positive bacteria and that the correlation with the ability to inhibit glycation is stronger (positive correlation, 0.8501) [55,56]. The correlation between the TA and the ability to inhibit glycation was strong (positive correlation, 0.9862), indicating that the roselle anthocyanin extract had a significant impact on the ability to inhibit glycation. However, although the correlation between the TP and TF was strong and positive (0.9891), the correlation between each of these values and the ability to inhibit glycation was negative (0.9918 and 0.9860, respectively). This result is different from that of a previous study and confirms that anthocyanins are the main factor affecting the antiglycation capacity. Anthocyanins terminate the glycation reaction by inhibiting glycoprotein synthesis, according to preliminary speculation [30]. The analysis results described above show that the TO content was higher in the ethanol extract (Table 2), which was strongly positively correlated with DPPH free radical scavenging ability (*R*^2^ = 0.8610) and the ability to inhibit glycation (*R*^2^ = 0.8501) (Figure 4), and these organic acids were effective at inhibiting both Gram-positive and Gram-negative bacteria (Table 3). The TA content was strongly positively correlated with the ability to inhibit glycation (*R*^2^ = 0.9862) (Figure 4).

**Figure 5:**
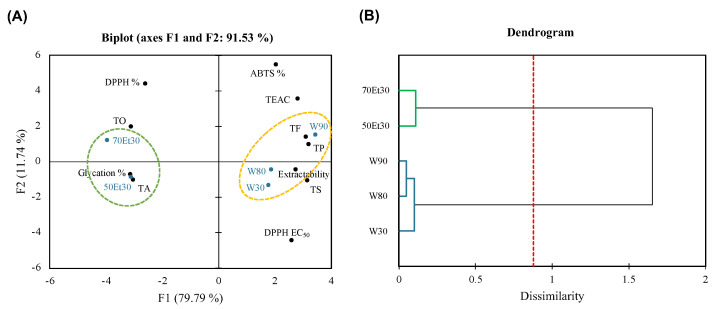
(**A**) Principal component analysis and (**B**) agglomerative hierarchical clustering of roselle extracts. Abbreviations: W30, W80, and W90 represent the samples extracted with water at 30, 80, and 90 °C, respectively; 50Et30 and 70Et30 represent the samples extracted with 0% and 70% ethanol, respectively; TS, TO, TA, TP, TF, DPPH%, ABTS%, and glycation% represent the total saccharides, total organic acids, total anthocyanins, total polyphenols, total flavonoids, DPPH radical scavenging for 1.75 mg/mL of extract, ABTS scavenging activity for 1.75 mg/mL of extract, and antiglycation capacity.

Figure 5 shows how the PCA and AHC were used to process the large dataset. A PCA often uses 2D or 3D modes to explain the correlation between samples or factors [57]. In this study, the 2D model had a data representativeness rate of 91.53%; that is, the sum of F1 (79.79%) and F2 (11.74%). This means that the data have high credibility and can clearly explain the differences between samples and correlations with various factors. By grouping the roselle extract samples through AHC, the water extract cluster (W30, W80, and W90) and ethanol extract cluster (50Et30 and 70Et30) were grouped due to significant differences between them (*p* < 0.05). Due to the higher extraction temperature of the water, the cluster is located on the right side of the PCA plot, showing higher extractability and TS, TP, and TF contents. The Pearson correlation analysis results showed that the ABTS free radical scavenging rate (ABTS %) was not highly correlated with the physical and chemical analysis factors described above (Figure 4), so we hypothesize that the higher TEAC and ABTS % in the water extraction group were due to extractability. Due to the lower extraction temperature of the ethanol, the cluster is located on the left side of the PCA plot and shows higher TO and TA values. Solvent solubility may have been the cause of the higher anthocyanin content. The results of the Pearson correlation analysis showed that the DPPH free radical scavenging rate (DPPH %), DPPH EC_50_, and ability to inhibit glycation (glycation %) were mainly characterized by a strong positive correlation between TO and TA (Figure 4). In this study, the differences in the characteristics of the extracts obtained using different solvents were greater than with different temperatures, which could inform the applications of different extraction methods.

## 4. Conclusions

This study clarified the significant impact of roselle extraction methods on functional properties. Water extraction demonstrated advantages in terms of the antioxidant capacity for ABTS free radicals, while ethanol extraction exhibited superior DPPH scavenging capacity, antiglycation activity, and bacterial growth inhibition. These results indicate the importance of solvent selection and the extraction conditions for optimizing the functional characteristics of roselle extracts. This study provides a novelty assessment model for functional roselle extraction selectivity, which may be applied for the rapid development of related products, such as alcoholic and non-alcoholic beverages.

## Figures and Tables

**Table 1:** The extractability and functional components of roselle extracts.

	Extractability	Total Saccharides	Total Organic Acids	Total Anthocyanins	Total Polyphenols	Total Flavonoids
Unit	%	mg/g				
50Et30	45.8 ± 3.7 ^b^	46.4 ± 0.5 ^b^	41.4 ± 0.1 ^a^	2.1 ± 0.1 ^a^	5.8 ± 0.3 ^c^	4.9 ± 0.6 ^b^
70Et30	42.4 ± 5.9 ^b^	-	44.1 ± 0.4 ^a^	1.8 ± 0.0 ^b^	6.0 ± 0.5 ^c^	5.5 ± 0.2 ^b^
W30	46.0 ± 4.4 ^ab^	98.0 ± 1.6 ^a^	32.3 ± 0.1 ^b^	1.1 ± 0.1 ^c^	10.5 ± 0.2 ^b^	8.8 ± 0.3 ^a^
W80	49.3 ± 2.7 ^a^	100.8 ± 0.4 ^a^	34.1 ± 0.6 ^b^	0.8 ± 0.0 ^d^	11.9 ± 0.4 ^a^	10.8 ± 0.2 ^a^
W90	49.2 ± 2.8 ^a^	124.1 ± 3.6 ^a^	33.2 ± 0.1 ^b^	0.8 ± 0.1 ^d^	13.0 ± 0.2 ^a^	11.4 ± 0.2 ^a^

Each value is expressed as the mean ± standard deviation (*n* = 3). Values (^a–d^) with different letters within the same column indicate significant differences (*p* < 0.05). Sample abbreviations: W30, W80, and W90 represent the samples extracted with water at 30, 80, and 90 °C, respectively; 50Et30 and 70Et30 represent the samples extracted with 0% and 70% ethanol, respectively.

**Table 2:** The antioxidant activity rates of the roselle extracts.

	TEAC (μM)	DPPH EC_50_ (μg/g)
50Et30	93.9 ± 3.5 ^d^	26.2 ± 1.7 ^b^
70Et30	102.6 ± 3.4 ^c^	21.1 ± 1.8 ^c^
W30	115.9 ± 4.5 ^b^	33.8 ± 2.3 ^a^
W80	109.8 ± 4.5 ^b^	28.7 ± 2.2 ^b^
W90	132.0 ± 3.4 ^a^	29.4 ± 2.3 ^b^

Each value is expressed as the mean ± standard deviation (*n* = 3). Values (^a–d^) with different letters within the same column indicate significant differences (*p* < 0.05). Abbreviations: W30, W80, and W90 represent the samples extracted with water at 30, 80, and 90 °C, respectively; 50Et30 and 70Et30 represent the samples extracted with 0% and 70% ethanol, respectively; TEAC and DPPH represented the Trolox equivalent antioxidant capacity and scavenging activity, respectively.

**Table 3:** The antibacterial capacity rates of roselle extracts.

	*Escherichia coli*		*Staphylococcus aureus*	
	MIC (mg/mL)	MBC (mg/mL)	MIC (mg/mL)	MBC (mg/mL)
50Et30	0.9 ^b^	0.9 ^b^	0.6 ^a^	1.0 ^b^
70Et30	1.0 ^a^	1.0 ^a^	0.6 ^a^	1.2 ^a^
W30	-	-	0.6 ^a^	1.2 ^a^
W80	-	-	-	-
W90	-	-	-	-

Each value is expressed as the mean ± standard deviation (*n* = 3). Values (^a,b^) with different letters within the same column indicate significant differences (*p* < 0.05). Abbreviations: W30, W80, and W90 represent the samples extracted with water at 30, 80, and 90 °C, respectively; 50Et30 and 70Et30 represent the samples extracted with 0% and 70% ethanol, respectively; MIC and MBC represent the minimum inhibitory concentration and minimum bactericidal concentration, respectively.

## Data Availability

The original contributions presented in the study are included in the article, further inquiries can be directed to the corresponding author.
